# Socioeconomic status and economic hardship attenuated the associations between early tobacco or nicotine use and brain outcomes in preadolescent children

**DOI:** 10.1515/nipt-2024-0022

**Published:** 2025-04-02

**Authors:** Pedro J. Rodriguez Rivera, Miriam S. Menken, William Chan, Amal Isaiah, Meghann C. Ryan, Christine C. Cloak, Thomas Ernst, Linda Chang

**Affiliations:** Department of Diagnostic Radiology and Nuclear Medicine, 12264 University of Maryland School of Medicine, Baltimore, MD, USA; Department of Otorhinolaryngology – Head and Neck Surgery, 12264 University of Maryland School of Medicine, Baltimore, MD, USA; Department of Pediatrics, 12264 University of Maryland School of Medicine, Baltimore, MD, USA; Department of Neurology, Johns Hopkins University School of Medicine, Baltimore, MD, USA; Department of Neurology, 12264 University of Maryland School of Medicine, Baltimore, MD, USA

**Keywords:** socioeconomic status, early tobacco/nicotine use, MRI, cognition

## Abstract

**Objectives:**

Early tobacco use (before age 11) is linked to poorer cognition and reduced cortical surface area and volume in young adolescents. This study examined how socioeconomic status (SES) factors – parental education, household income, and economic hardships – influenced these associations.

**Methods:**

Using baseline (N=11,876) and year 3 (N=10,414) datasets from the Adolescent Brain Cognitive Development study, we assessed the impact of baseline tobacco/nicotine use initiation on cognitive scores, cortical volume, thickness, and surface area across the entire cohort and in propensity-matched subgroups. Linear mixed effects models controlled for SES and other covariates, with multiple comparison correction. Analyses were cross-sectional at baseline and longitudinal with both timepoints.

**Results:**

Compared to non-users (N=11,240), early users (N=110) had more advanced pubertal development (p=0.003) and economic hardships (p<0.001), but fewer girls (p=0.04), Hispanics (p=0.02), parents with graduate degrees (p<0.001) and high-income families >$100 K (p=p<0.001). Relative to non-users, early users had poorer cognitive scores (Cohen’s *d*: −0.69 to −0.24), smaller surface areas (Cohen’s *d*: −2.28 to −0.22), similar cortical thickness at both timepoints, and by year 3, smaller cortical volumes (Cohen’s *d*: −1.06 to −1.24). However, SES-adjustments eliminated cognitive scores and volumes differences and reduced cortical surface area effects at both timepoints (Cohen’s *d*: −1.92 to −0.51). After propensity score matching, early users and non-users showed no cognitive or brain differences, regardless of SES adjustments.

**Conclusions:**

Adjusting for SES eliminated the negative impact of early tobacco/nicotine use on cognition and reduced its effect on brain surface area, underscoring the importance of SES in morphometry studies.

## Introduction

Adolescent tobacco and nicotine use negatively impacts neurodevelopment [1], including abnormalities on cognition and brain morphometry in young preadolescents who initiated tobacco use [2]. Tobacco/nicotine use is also more prevalent among individuals from lower socioeconomic backgrounds, as assessed by family income and parental education [3], [4], [5]. In the large longitudinal Adolescent Brain Cognitive Development (ABCD) Study, higher socioeconomic status (SES) consistently correlated with better cognitive performance and more advanced brain structure [6], [7], [8]. Given the interplay between SES, tobacco/nicotine use, and neurodevelopment, examining these relationships is essential.

Parental education and family income are common indicators of SES, shaping family dynamics, parent-child relationships, and neurodevelopment [6], 7], [9], [10], [11]. Higher parental education provides cognitive stimulation and enrichment opportunities that enhance cognitive and academic outcomes [12], 13]. Conversely, lower education may limit these opportunities, affecting neurodevelopment. Family income reflects material resources that support cognitive and socioemotional growth, including healthcare and educational access [14], 15]. Higher income also correlates with enriching environments and extracurricular activities critical for brain development [14], 15]. However, research often uses categorical income measures that fail to capture diverse lived experiences across SES contexts [16], [17], [18]. Assessing economic hardship alongside income is crucial for understanding SES effects on cognition and neurodevelopment. It acknowledges that even high-income families may face financial strain from large households or significant obligations.

This study aims to assess how SES – specifically parental education, family income, and economic hardships – shapes the relationship between early tobacco/nicotine use (first use before age 11 years) and cognitive measures or brain morphometry in the ABCD Study cohort. We additionally applied propensity score matching to separate SES effects from those of early tobacco/nicotine use, balancing comparisons between early-user and non-user groups across demographics. We hypothesized that early tobacco/nicotine use would correlate with poorer cognitive measures and abnormalities on cortical morphometry but that SES variables would attenuate the effects on these associations.

## Methods

### Data source

We used data from the ABCD Study, baseline and 2-year follow-up (Year 3) visits from the National Institutes of Mental Health Data Archive (v.4.0). The ABCD study is a 10-year follow-up of 11,876 children enrolled at 9–10 years old, along with their participating parent/caregivers, across 21 sites in the United States. The baseline ABCD dataset was collected between 2016 and 2018 and the year 3 dataset was collected between 2018 and 2021. At each visit, participants provided written informed consent or assent, and all protocols were approved by the central (UCSD) and site-specific Institutional Review Boards. Recruitment, inclusion/exclusion, and site selection are well-documented, and the study sample size has sufficient power to detect mall to medium effect sizes [8].

### Measures

#### Early tobacco or nicotine use

We used self-reported substance use data collected at the baseline visit [19]. Participants were classified as ‘early-users’ if they reported any tobacco/nicotine use at baseline, and ‘non-users’ if they reported none.

This initial classification was maintained for longitudinal analyses. However, children who began using tobacco/nicotine after the baseline visit were not reclassified as ‘early-users.’ Instead, they were included in a separate covariate labeled ‘other substances used’. This covariate also accounted for participants who reported the use of any other substances (e.g., alcohol, marijuana) in addition to tobacco/nicotine use since baseline.

#### Cognitive measures

We evaluated a set of cognitive measures derived from the NIH Cognition Battery Toolbox^®^ [20]. At baseline, seven uncorrected test scores were obtained to assess flexible thinking, cognitive control, attention, working memory, reading, processing speed, visuospatial memory, and language/verbal intellect. Three composite scores were generated: Crystallized Cognition (language/verbal intellect and reading), Fluid Composite (from the other five test scores), and Cognitive Total Composite (all seven scores). At year 3, only five uncorrected scores and one composite scores were obtained; since the Dimensional Card Change Sort and List Sorting Working Memory tasks were not administered, the Fluid and Total Composite scores could not be generated.

#### Magnetic resonance imaging (MRI) measures

T1-weighted (1-mm isotropic resolution) structural magnetic resonance imaging (MRI) scans were acquired using a harmonized protocol on 3T Siemens Prisma, Philips, or General Electric 750 MR scanners [21]. Automated reconstruction and segmentation of the baseline and second follow-up scans were performed by the ABCD Data Analysis, Informatics and Resource Center using FreeSurfer v5.3. The segmented imaging data for our analyses were processed using the Desikan-Killiany atlas [22], comprising cortical surface area, thickness and volumes of the whole brain, as well as 34 brain regions [21].

#### Covariates and other factors including economic hardship

We evaluated caregiver-reported youth age, sex assigned at birth, and race/ethnicity (Table 1). Additionally, we assessed baseline youth self-reported Pubertal Stage, Menstrual Phase [23], Parental Monitoring [24], and School Risk and Protective Factors [25]. Higher scores indicated greater pubertal development, stronger parental monitoring, and a more favorable school environment. SES variables included parent-reported education level, annual household income, and economic hardships. As previously described [26], economic hardship was derived from seven caregiver-reported items in the ABCD Demographics Questionnaire, assessing material or financial deprivation, including inability to afford food, loss of telephone service, unpaid rent/mortgage, eviction risk, utility suspensions, or inability to access medical/dental care.

**Table 1:** Participant characteristics in the ABCD study.

Participant characteristics	Cognitive analyses (N=11,242)			MRI analyses (N=11,350)		
	Early-users (N=108)	Non-users (N=11,134)	p-Value	Early-users (N=110)	Non-users (N=11,240)	p-Value
**Age (in months), mean (SD)**	120 (7.92)	119 (7.49)	0.18	120 (7.92)	119 (7.48)	0.12
**Pubertal developmental stage, mean (SD)**	2.31 (0.859)	2.03 (0.850)	<0.001	2.27 (0.845)	2.03 (0.851)	0.003
**Sex, n (%)**			0.03			0.04
Boys	68 (63.0 %)	5,814 (52.2 %)		69 (62.7 %)	5,875 (52.3 %)	
Girls	40 (37.0 %)	5,320 (47.8 %)		41 (37.3 %)	5,365 (47.7 %)	
**Youth’s race, n (%)**			0.02			0.02
White	55 (50.9 %)	5,907 (53.1 %)		56 (50.9 %)	5,969 (53.1 %)	
Black	17 (15.7 %)	1,608 (14.4 %)		18 (16.4 %)	1,610 (14.3 %)	
Hispanic	13 (12.0 %)	2,233 (20.1 %)		13 (11.8 %)	2,255 (20.1 %)	
Other races	23 (21.3 %)	1,386 (12.4 %)		23 (20.9 %)	1,406 (12.5 %)	
**Parental education, n (%)**			<0.001			<0.001
High school or less	16 (14.8 %)	1,534 (13.8 %)		17 (15.5 %)	1,549 (13.8 %)	
AA or some college	54 (50.0 %)	2,861 (25.7 %)		53 (48.2 %)	2,886 (25.7 %)	
Bachelors	23 (21.3 %)	2,869 (25.8 %)		24 (21.8 %)	2,893 (25.7 %)	
Graduate or higher	15 (13.9 %)	3,870 (34.8 %)		16 (14.5 %)	3,912 (34.8 %)	
**Family income, n (%)**			<0.001			<0.001
Less than $50,000	56 (51.9 %)	3,533 (31.7 %)		56 (50.9 %)	3,562 (31.7 %)	
$50,000 to $99,999	35 (32.4 %)	3,060 (27.5 %)		37 (33.6 %)	3,090 (27.5 %)	
$100,00 or higher	17 (15.7 %)	4,541 (40.8 %)		17 (15.5 %)	4,588 (40.8 %)	
**Economic hardships, n (%)**			<0.001			<0.001
Yes	49 (45.4 %)	2,375 (21.3 %)		49 (44.5 %)	2,388 (21.2 %)	
No	59 (54.6 %)	8,759 (78.7 %)		61 (55.5 %)	8,852 (78.8 %)	
**Other drugs use, n (%)**			<0.001			<0.001
Yes	63 (58.3 %)	2,504 (22.5 %)		66 (60.0 %)	2,511 (22.3 %)	
No	45 (41.7 %)	8,630 (77.5 %)		44 (40.0 %)	8,729 (77.7 %)	
**School environment, mean (SD)**	3.15 (0.565)	3.32 (0.467)	<0.001	3.15 (0.561)	3.32 (0.468)	<0.001
**Parental monitoring, mean (SD)**	4.12 (0.650)	4.39 (0.511)	<0.001	4.12 (0.650)	4.39 (0.510)	<0.001

Values are presented as mean (SD) for continuous variables and n (%) for categorical variables. SD refers to standard deviation. p-values were calculated using chi-square tests for categorical variables and unpaired Student’s *t*-tests for continuous variables. ‘Early-users’ are children who reported any tobacco/nicotine use at baseline (before age 11), while non-users reported no use. Percentages represent column proportions.

#### Statistical analysis

We compared sociodemographic variables between baseline early-users and non-users using Chi-squared tests, odds ratios, and unpaired *t*-tests as appropriate. In the cross-sectional model, the impact of baseline tobacco/nicotine use on baseline cognitive measures and brain morphometry was evaluated using linear mixed effects models (LMEs). The initial cross-sectional models included the following fixed effects: youth’s sex assigned at birth, race/ethnicity, age (in months), other substances used (as previously defined), pubertal development, parental monitoring, school environment, and handedness, with intracranial volume included for volumetric brain measures. Family ID was used as a random effect for cognitive measures, while scanner manufacturer was used as a random effect for morphometric brain measures. The SES-adjusted cross-sectional models additionally included family income, economic hardships, and caregiver education as fixed effects.

For longitudinal analyses, we fitted LMEs to assess the effects of baseline tobacco/nicotine use, visit, and their interaction as fixed effects on uncorrected cognition scores and cortical surface area, thickness, and volumes across both time points: baseline and Year 3 (2-Year Follow-up). For cognitive measures, both Participant and Family IDs were random effects. For morphometric measures, Participant ID was a random effect, and scanner manufacturer was a fixed effect. All covariates from the cross-sectional models were also included in the longitudinal models, both with and without SES adjustment. After running initial models with the tobacco-by-visit interaction, non-significant interaction terms were removed. Cohen’s *d* effect sizes were derived for the main effects (e.g., early use in cross-sectional analyses and early use, visit, and their interaction in longitudinal analyses), with false discovery rate (FDR) correction applied, setting significance at p<0.05.

To assess the robustness of our findings and mitigate confounding due to baseline group imbalances and differences across the two time points, we conducted post-hoc propensity score matching (PSM). Participants were matched 1:1 using nearest neighbor matching without replacement based on sex, age, and race/ethnicity – key demographic factors influencing both substance use and neurodevelopment. SES variables (parental education, household income and experiencing economic hardships) were excluded from matching to preserve variability and assess their moderating effects but were included as fixed effects in SES-adjusted models. Pubertal development was also excluded from matching to avoid overmatching, following methodological recommendations to omit potential mediators from the matching process [27], 28]; however, it was included as a fixed effect in LMEs to adjust for potential confounding. Standardized mean differences (SMD<0.1) confirmed successful balance post-matching, and LMEs were refitted using the matched samples to evaluate whether observed associations remained consistent with and without SES adjustment. All statistical analyses were performed in R version 4.0.3, with LMEs and effect sizes generated using lme4 [29] and emmeans packages [30], propensity score matching using the MatchIt package [31], and brain maps generated using the ggseg3d package [32].

## Results

### Prevalence of tobacco/nicotine use in the ABCD study baseline cohort

Of the 11,876 baseline participants, 634 were excluded from cognitive analyses (final N=11,242) and 526 from MRI analyses (final N=11,350) due to missing data (Table 1). Among the remaining participants, 108 early-users and 11,134 non-users were identified in the cognitive dataset, and 110 early-users and 11,240 non-users in the imaging dataset.

**Figure 1:**
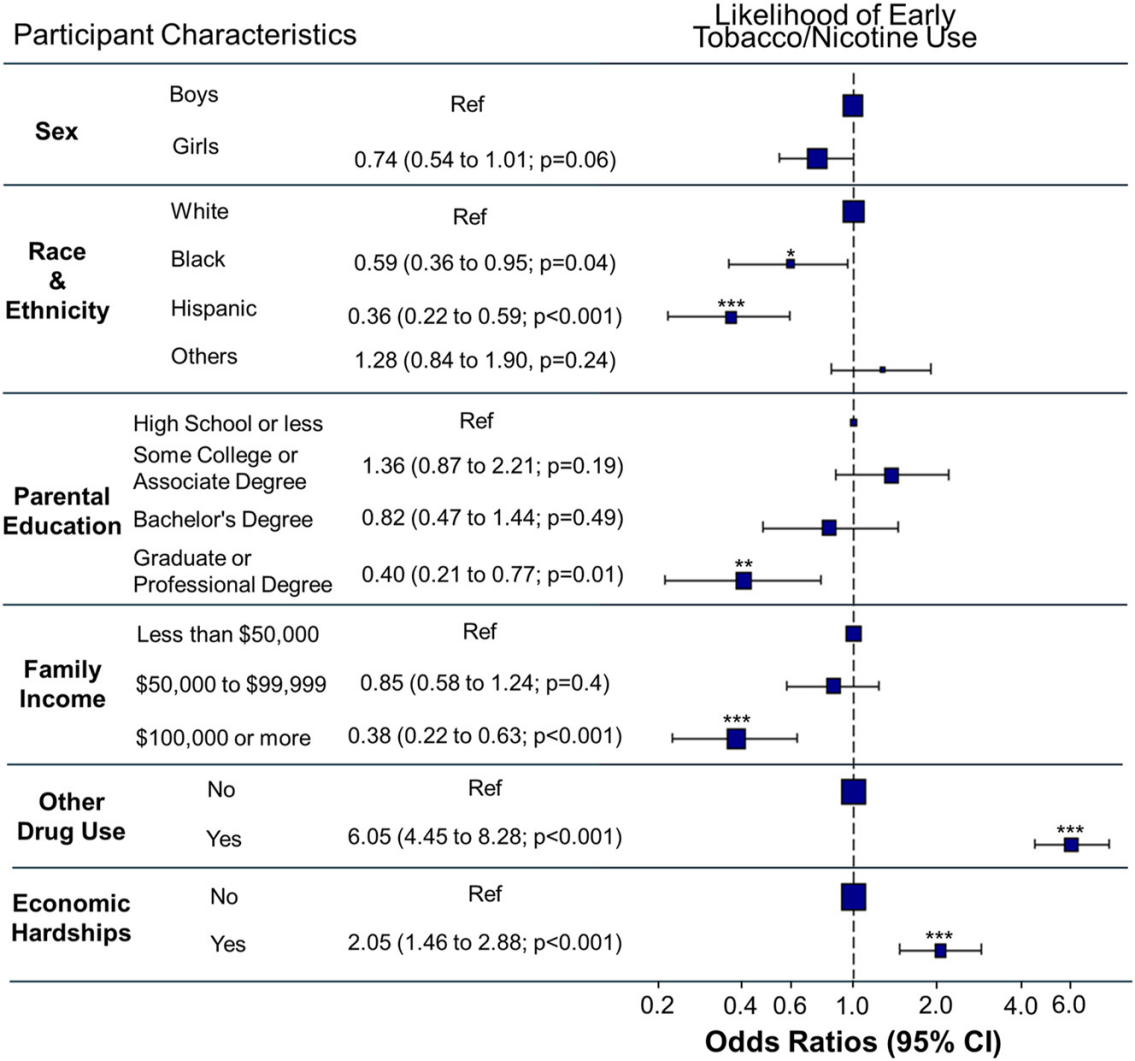
Odds ratios for early tobacco or nicotine use. This plot displays the odds ratios for the likelihood of early tobacco/nicotine use based on various participant characteristics. Early use refers to children who reported any tobacco/nicotine use at the baseline visit (before age 11). Asterisks denote the significance of odds ratios, where * = p-value <0.05, ** = p-value <0.01 and *** = p-value <0.001.

Early users showed more advanced pubertal development, were less likely to be Hispanic, and more likely to identify as Other races compared to non-users. Parents of early users were less likely to have education beyond an Associate’s or college degree (p<0.001), more likely to report family incomes <$50,000, and less likely to report incomes >$100,000 (p<0.001). Early users also experienced greater financial hardship (p<0.001) and were more likely to use other drugs (p<0.001), report poorer school environments (p<0.001), and experience less parental monitoring (p<0.001) than non-users (Figure 1). Additionally, children from higher-income families (>$100,000) were less likely to be early users than those from lower-income families (Figure 1; OR for $50–$100,000: 0.85, 95 % CI [0.58, 1.24], p=0.4; >$100,000: OR, 0.38, 95 % CI [0.22, 0.63], p<0.001).

Despite a higher proportion of Black (69.1 %) and Hispanic (52.7 %) participants in lower-income families (<$50,000) compared to Whites (14 %; p<0.001), Black and Hispanic children were less likely than Whites to initiate tobacco/nicotine use at ages 9–10 years (Figure 1; Black: OR, 0.59, 95 % CI [0.36, 0.95], p=0.04; Hispanic: OR, 0.36, 95 % CI [0.22, 0.59], p<0.001).

### Cognitive scores and tobacco/nicotine use in relation to SES factors

#### Cross-sectional analysis without SES

**Figure 2:**
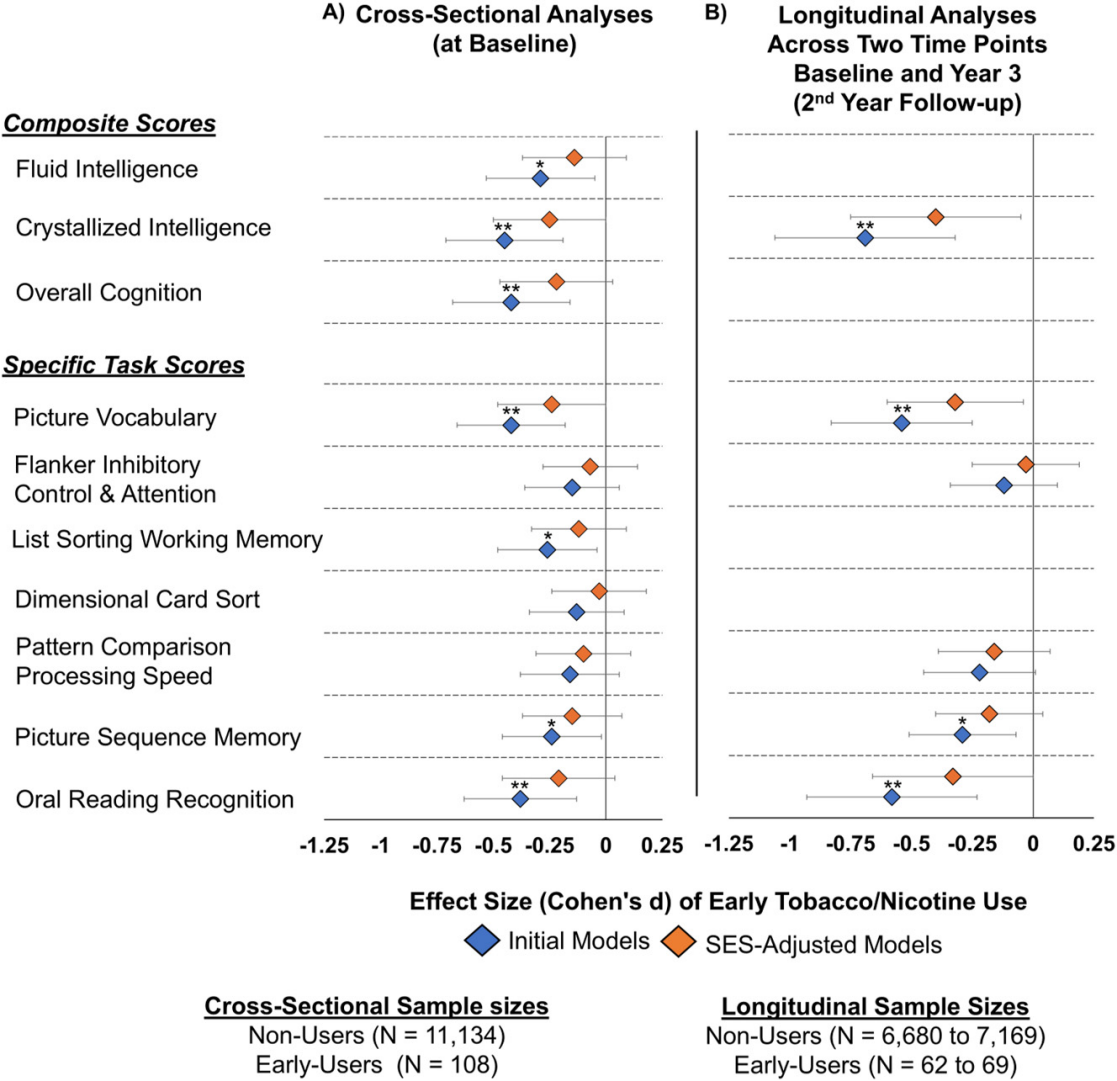
Association between early tobacco or nicotine use and cognitive scores, before and after adjustment for socioeconomic status (SES) variables). (A) Displays the cross-sectional effect sizes (Cohen’s *d*) and 95 % confidence intervals of early tobacco/nicotine use on cognitive scores, before (initial models) and after adjustment for socioeconomic status (SES) variables (SES-adjusted models). (B) Displays the longitudinal effect sizes. “Early-users” refers to children who reported any tobacco/nicotine use at the baseline visit (before age 11), while non-users are those who did not report any use. Effect sizes were derived using linear mixed models (LMEs) and estimated marginal means. Uncorrected t-scores from the NIH Toolbox were used for these analyses. The initial models included fixed effects for early tobacco use, child’s age, sex, race, ever use of other substances, and scores of pubertal developmental stage, parental monitoring, and school risk and environment. The SES-adjusted models included all fixed effects from the initial models, along with SES variables: average annual family income, parental education, and history of economic hardships. Family IDs were included as random effects in cross-sectional analyses, while both Family and participant IDs were included as random effects in the longitudinal analyses. Negative effect sizes indicate lower cognitive scores for early users compared to non-users. Asterisks in (A) and (B) denote the significance of effect sizes before and after SES adjustment in the LMEs: * = p-value <0.05 and ** = p-value <0.01. Longitudinal effect sizes for the List Sorting Working Memory, Dimensional Card Sort tasks, and composite scores are absent as these tasks were not administered or derived at year 3 (2nd year follow-up).

Initial models showed that the children who reported early tobacco or nicotine use had lower cognitive scores than non-users on Picture Vocabulary (Cohen’s *d*: −0.42, 95 % CI [−0.66, −0.18]; p=0.003), List Sorting Working Memory (Cohen’s *d*: −0.26, 95 % CI [−0.48, −0.04]; p=0.037), Picture Sequence Memory (Cohen’s *d*: −0.24, 95 % CI [−0.46, −0.18]; p=0.043), and Oral Reading Recognition (Cohen’s *d*: −0.38, 95 % CI [−0.64, −0.13]; p=0.007). Relative to non-users, these early users also had lower scores on Fluid Composite (Cohen’s *d*: −0.29, 95 % CI [−0.52, −0.05]; p=0.036), Crystallized Cognition (Cohen’s *d*: −0.45, 95 % CI [−0.71, −0.19]; p=0.003), and Total Cognition (Cohen’s *d*: −0.42, 95 % CI [−0.67, −0.16]; p=0.003) (Figure 2A, Table S1).

#### Longitudinal analysis without SES

Early users also had lower scores than non-users across both time points (baseline and Year 3) in Picture Vocabulary (Cohen’s *d*: −0.54, 95 % CI [−0.83, −0.25]; p=0.001), Picture Sequence Memory (Cohen’s *d*: −0.29, 95 % CI [−0.51, −0.07]; p=0.016), and Oral Reading Recognition (Cohen’s *d*: −0.58, 95 % CI [−0.92, −0.23]; p=0.002), as well as lower Crystallized Cognition scores (Cohen’s *d*: −0.69, 95 % CI [−1.06, −0.32]; p=0.001) (Figure 2B, Table S2).

#### Impact of SES factors on cross-sectional and longitudinal cognitive performance

**Figure 3:**
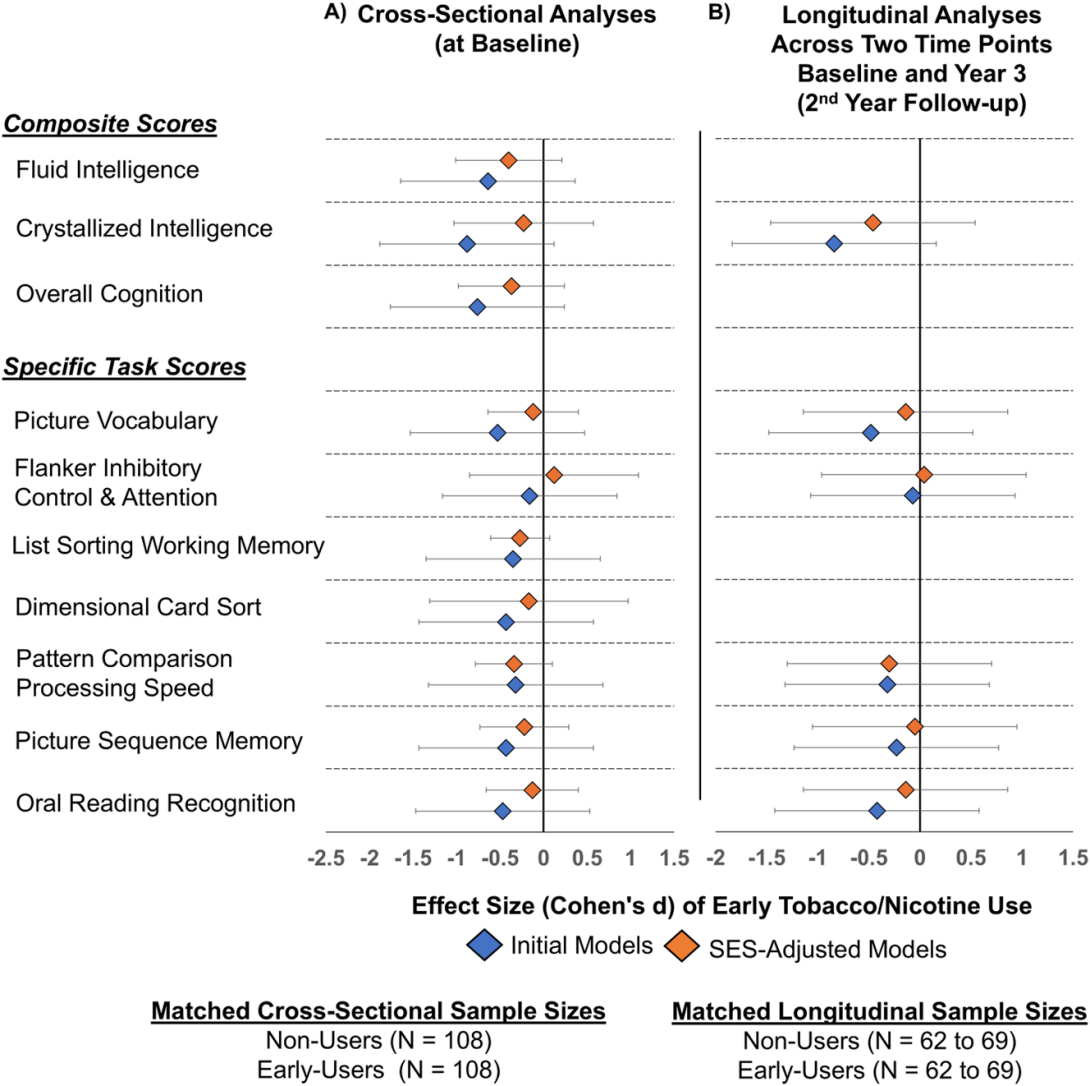
Association between early tobacco or nicotine use and cognitive scores post propensity score matching, before and after adjustment for socioeconomic status (SES) variables. (A) Cross-sectional effect sizes (Cohen’s *d*) and 95 % confidence intervals for the association between early tobacco/nicotine use and cognitive scores, before adjustment (initial models) and after adjustment for SES variables (SES-adjusted models). (B) Longitudinal effect sizes under the same conditions. “Early-users” refers to participants who reported any tobacco/nicotine use at the baseline visit (before age 11), while non-users are those who did not report any use. Propensity score matching balanced early-users and non-users by age, sex, and race/ethnicity, resulting in a matched sample of N=108 at baseline for cross-sectional analyses and N=69 at year 3 (2nd year follow-up) for longitudinal analyses. Seven early users had missing crystallized composite scores at follow-up, reducing the sample size for this measure to N=62. Effect sizes were derived using linear mixed models (LMEs) and estimated marginal means. Uncorrected t-scores from the NIH Toolbox were used for these analyses. Initial models included fixed effects for early tobacco/nicotine use, ever use of other substances, pubertal development, parental monitoring, and school risk/environment. The child’s age, sex and race/ethnicity were not included as they were covariates in the matching process. The SES-adjusted models included all fixed effects from the initial models, along with SES variables: average annual family income, parental education, and history of economic hardships. Family IDs were included as random effects in cross-sectional analyses, while both Family and participant IDs were included as random effects in the longitudinal analyses. Negative effect sizes indicate lower cognitive scores for early users compared to non-users. Longitudinal effect sizes for the List Sorting Working Memory, Dimensional Card Sort tasks, and composite scores are absent as these tasks were not administered or derived at year 3 (2nd year follow-up).

Both the cross-sectional (baseline) and longitudinal associations with early tobacco use were no longer significant after adjusting for SES variables (Figure 2A and B, Tables S1 and S2). Furthermore, in both the original and SES-adjusted models, no significant interactions between early use and visit were observed (results not shown). Similarly, the post-hoc analyses using propensity score matching revealed no group differences in cognitive scores between early-users and non-users, either cross-sectionally at baseline or over the two-year period (Figure 3, Tables S3 and S4).

### Cortical surface area and tobacco/nicotine use in relation to SES factors

#### Cross-sectional analysis without SES

**Figure 4:**
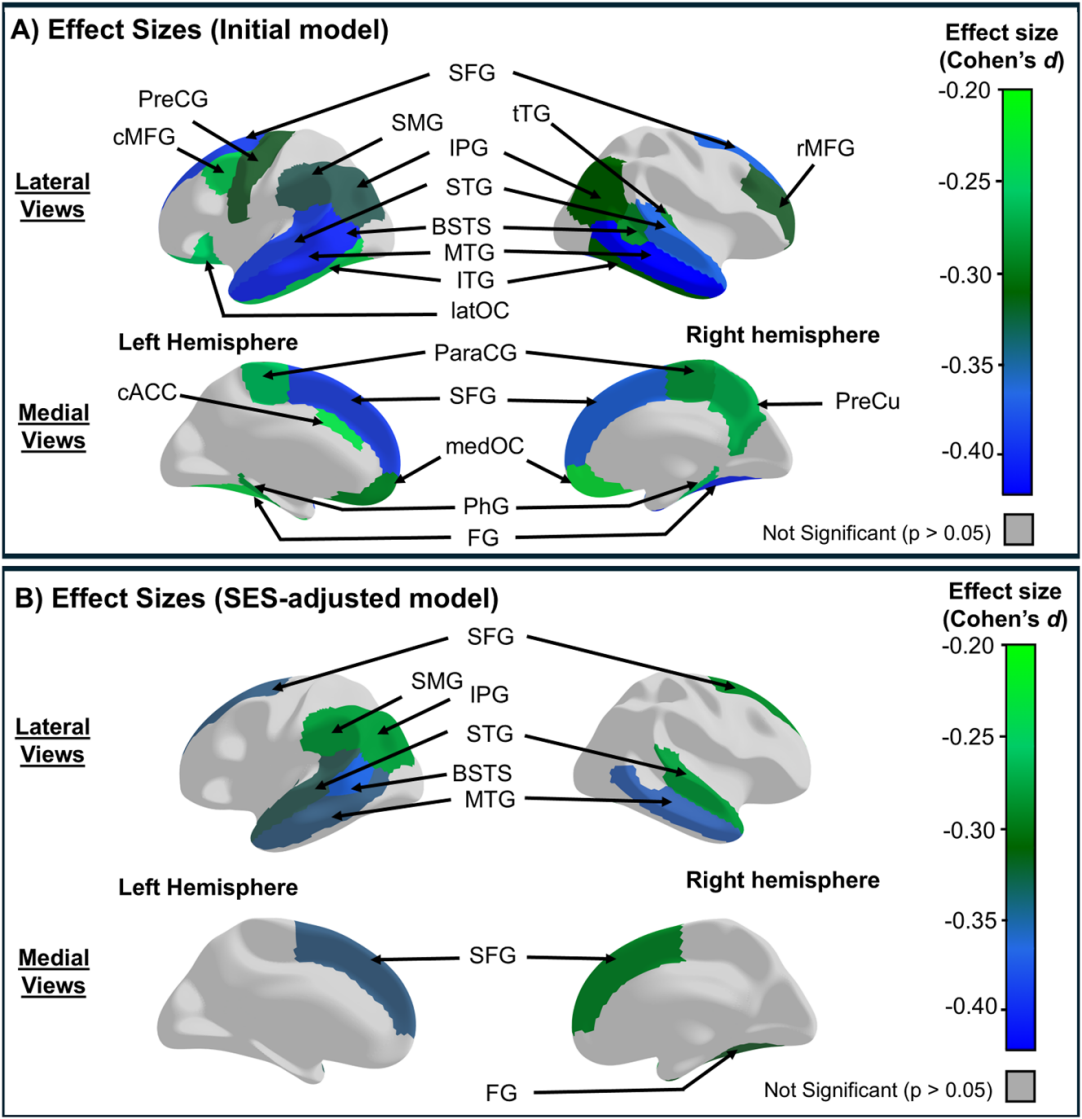
Cross-sectional group differences in cortical surface area between early tobacco or nicotine users and non-users at baseline. (A) Shows the region-specific cortical surface areas with significant cross-sectional effect sizes (Cohen’s *d*) of early tobacco/nicotine use before adjusting for socioeconomic status (SES) variables. (B) Shows the region-specific cortical surface areas with significant effect sizes after adjusting for SES variables. “Early-users” refers to participants who reported any tobacco/nicotine use at the baseline visit (before age 11), while non-users are those who did not report any use. Effect size estimates were derived using linear mixed models (LMEs) and estimated marginal means. The initial models included fixed effects for early tobacco/nicotine use, child’s age, sex, race, ever use of other substances, handedness, and scores of pubertal developmental stage, parental monitoring, and school risk and environment. The SES-adjusted models included all fixed effects from the Initial models, along with SES variables: average annual family income, parental education, and history of economic hardships. Scanner manufacturer was included as random effects in both models. Negative effect sizes indicate smaller surface areas in early-users compared to non-users. Significance was set at p-values <0.05 after correcting for multiple comparisons using the False Discovery Rate (FDR) approach. BSTS, banks of the superior temporal sulcus; cACC, caudal anterior cingulate; cMFG, caudal middle frontal; FG, fusiform; IPG, inferior parietal; ITG, inferior temporal; latOC, lateral orbitofrontal; medOC, medial orbitofrontal; MTG, middle temporal; PhG, parahippocampal; ParaCG, paracentral; PreCG, precentral; PreCu, precuneus; rMFG, rostral middle frontal; SFG, superior frontal; STG, superior temporal; SMG, supramarginal; tTG, transverse temporal.

At baseline, early-users had smaller whole-brain surface areas compared to non-users, with Cohen’s *d*: −0.34, 95 % CI [−0.53, −0.14] and significance levels from p<0.001 to p=0.005 (Table S5). Additionally, specific brain regions in the early users, including five in the left hemisphere, three in the right, and ten bilaterally, also showed smaller surface areas than non-users, with effect sizes ranging from Cohen’s *d*: −0.42 to −0.22 and significance levels from p<0.001 to p=0.049 (Figure 4A, Table S5).

#### Cross-sectional analysis with SES

After SES adjustments, the supramarginal gyrus in the left hemisphere as well as the middle temporal, superior frontal, and superior temporal gyri continued to show a smaller surface area in early-users than non-users. However, the group differences in other regions differed between the models. Prior to SES adjustment, both hemispheres of the banks of the superior temporal sulcus and the inferior parietal gyri showed group differences; after SES-adjustment, smaller surface areas in the early-users than non-users were observed solely in the left hemisphere. Conversely, after SES-adjustment, the smaller surface areas in bilateral fusiform cortices became smaller only in the right hemisphere in the early-users than non-users. These models including SES resulted in effect sizes ranging from Cohen’s *d*: −0.35 to −0.27, with significance levels from p<0.001 to p=0.035 (Figure 4B, Table S5).

**Figure 5:**
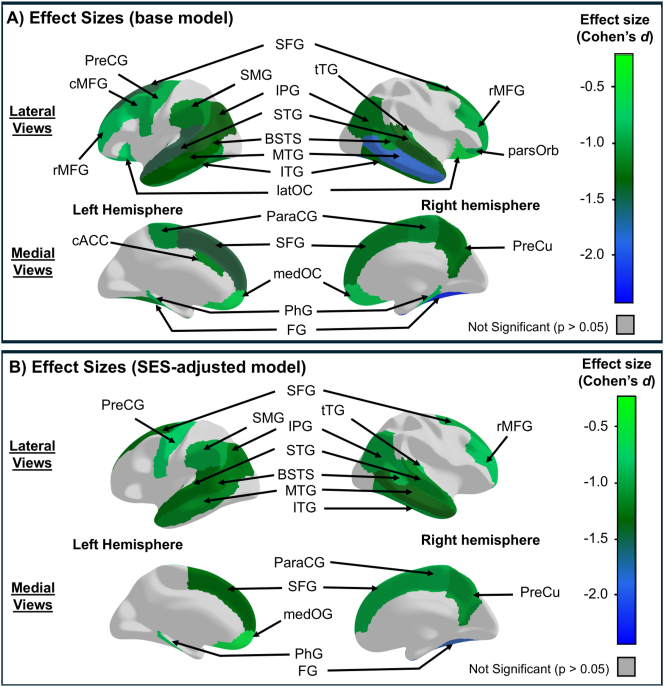
Longitudinal differences in cortical surface area between early tobacco/nicotine users and non-users across baseline and year 3 (2nd year follow-up). (A) Shows the region-specific cortical surface areas with significant longitudinal effect sizes (Cohen’s *d*) of tobacco/nicotine use before adjusting for socioeconomic status (SES) variables. (B) Shows the region-specific cortical surface areas with significant longitudinal effect sizes after adjusting for SES variables. “Early-users” refers to participants who reported any tobacco/nicotine use at the baseline visit (before age 11), while non-users are those who did not report any use. Effect size estimates were derived using linear mixed models (LMEs) and estimated marginal means. The initial models included fixed effects for tobacco/nicotine use at baseline, child’s age, sex, race, ever use of other substances, handedness, and scores of pubertal developmental stage, parental monitoring, school risk and environment, and scanner manufacturer. The SES-adjusted models included all fixed effects from the initial models, along with SES variables: average annual family income, parental education, and history of economic hardships. Scanner manufacturer was included as a random effect in cross-sectional analyses, whereas it was included as a fixed effect with participant IDs as random effects in longitudinal analyses. Negative effect sizes indicate smaller surface areas in early-users compared to non-users. Significance was set at p-values <0.05 after correcting for multiple comparisons using the False Discovery Rate (FDR) approach. BSTS, banks of the superior temporal sulcus; cACC, caudal anterior cingulate; cMFG, caudal middle frontal; FG, fusiform; IPG, inferior parietal; ITG, inferior temporal; latOC, lateral orbitofrontal; medOC, medial orbitofrontal; MTG, middle temporal; PhG, parahippocampal; ParaCG, paracentral; parsOrb, pars orbitalis; PreCG, precentral; PreCu, precuneus; rMFG, rostral middle frontal; SFG, superior frontal; STG, superior temporal; SMG, supramarginal; tTG, transverse temporal.

#### Longitudinal analysis without SES

The longitudinal models showed smaller surface areas in 6 of the 8 brain regions that previously showed unilateral group differences in the initial cross-sectional models. Additionally, a new region, the surface area of the right pars orbitalis was smaller in early-users than non-users and was observed only in the longitudinal model. Furthermore, two of the original eight regions – the rostral middle frontal gyrus and lateral orbitofrontal cortex – which previously showed unilateral differences in the cross-sectional analysis, now exhibited smaller surface areas bilaterally in early-users compared to non-users. Overall, the initial longitudinal models identified smaller surface areas in early-users than non-users in 19 regions: four in the left hemisphere, three in the right, and twelve bilaterally, with effect sizes ranging from Cohen’s *d* = −2.28 to −0.52 and significance levels from p<0.001 to p=0.045 (Figure 5A, Table S6).

**Figure 6:**
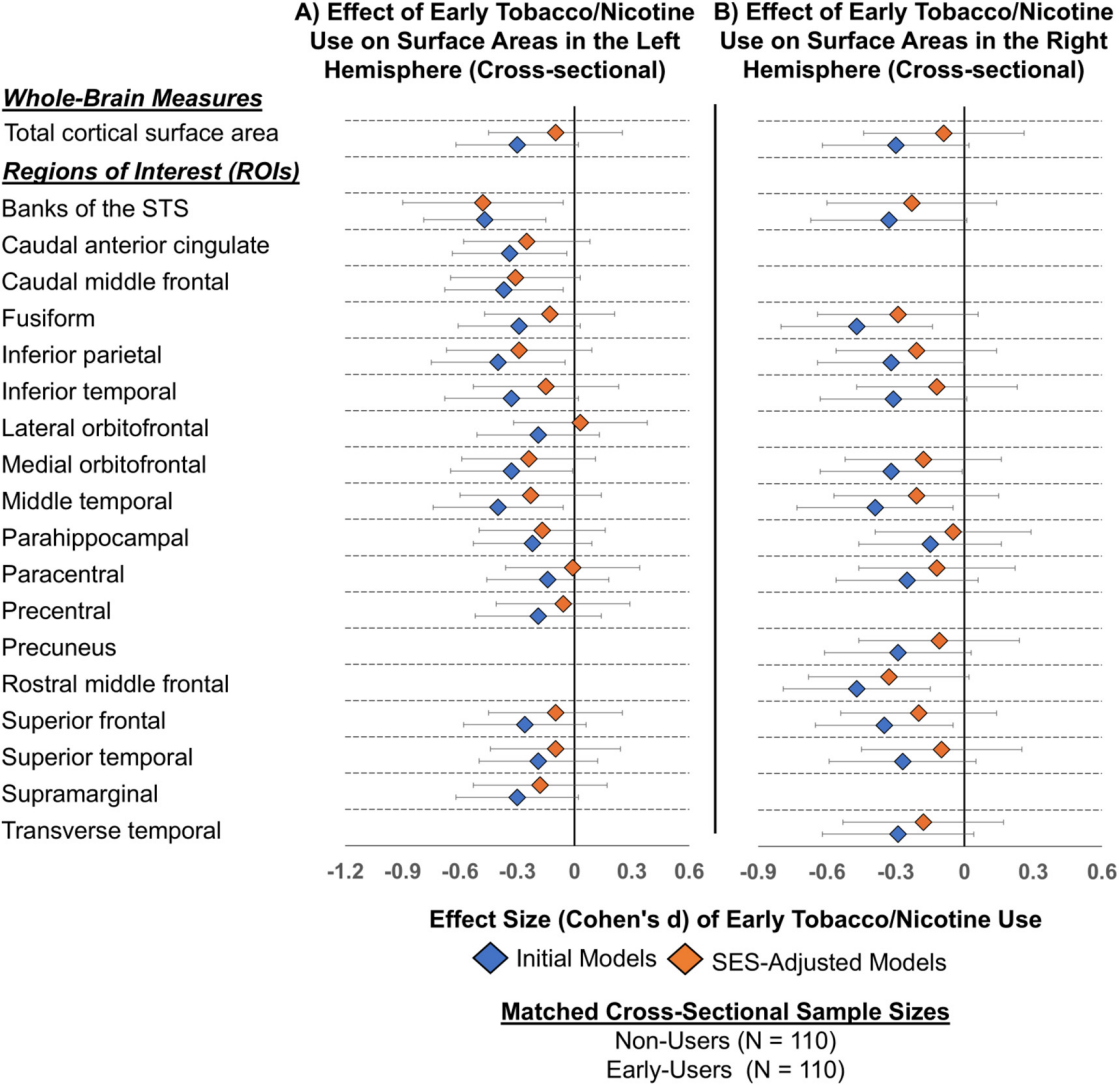
Cross-sectional association between early tobacco or nicotine use and cortical surface area post propensity score matching, before and after adjustment for socioeconomic status (SES) variables. (A) Cross-sectional effect sizes (Cohen’s *d*) and 95 % confidence intervals for the association between early tobacco/nicotine use and cortical surface area across regions of interest (ROIs) in the left hemisphere, before adjustment (initial models) and after adjustment for SES variables (SES-adjusted models). (B) Results for ROIs in the right hemisphere. Propensity score matching balanced early-users and non-users by age, sex, and race/ethnicity, resulting in a matched sample of **N=110** at baseline for cross-sectional analyses. The whole-brain and ROI-based surface areas shown in the plot include only regions that were significantly associated with early tobacco/nicotine use before propensity score matching (shown in Figure 4 and Table S5). Effect sizes were derived using linear mixed models (LMEs) and estimated marginal means. The initial model included fixed effects for early tobacco/nicotine use, ever use of other substances, pubertal development, parental monitoring, school risk/environment, and scanner manufacturer. The child’s age, sex and race/ethnicity were not included as they were covariates in the matching process. The SES-adjusted model included all variables from the initial model, plus socioeconomic status (SES) variables: average annual family income, parental education, and economic hardships. Participant IDs were included as random effects in both models (i.e., initial and SES-adjusted). Negative effect sizes indicate smaller cortical surface areas for “early-users” compared to non-users. Empty spaces indicate ROIs that were not significantly associated with early tobacco/nicotine use before the propensity score matching process. Banks of the STS, banks of the superior temporal sulcus.

#### Longitudinal analysis with SES

SES-adjusted models showed that compared to non-users, early users continued to have smaller surface areas across baseline and 2-year follow-up in the left precentral (Cohen’s *d*: −0.82, 95 % CI [−1.42, −0.22]; p=0.035) and left supramarginal (Cohen’s *d*: −1.08, 95 % CI [−1.86, −0.30]; p=0.033) gyri, the right precuneus (Cohen’s *d*: −1.18, 95 % CI [−2.14, −0.23]; p=0.048) and right transverse temporal gyrus (Cohen’s *d*: −0.92, 95 % CI [−1.64, −0.19]; p=0.046) (Figure 5B, Table S6). Additionally, five regions continued to exhibit smaller surface areas in the early users than non-users bilaterally with effect sizes ranging from Cohen’s *d*: −1.48 to −1.43 and significance from p<0.001 to p=0.014 (Figure 5B, Table S6).

Despite SES adjustments, longitudinal analyses continue to reveal smaller whole-brain surface areas over time (Cohen’s *d*: −1.72, 95 % CI [−2.84, −0.6] and significance levels from p=0.014 to p=0.019) (Table S6) in early-users compared to non-users. Additionally, SES-adjusted models indicated changes in the pattern of longitudinal associations. Six regions that showed bilateral effects linked to early use now exhibited smaller surface areas only and unilaterally following SES adjustments (Figure 5B, Table S6).

**Figure 7:**
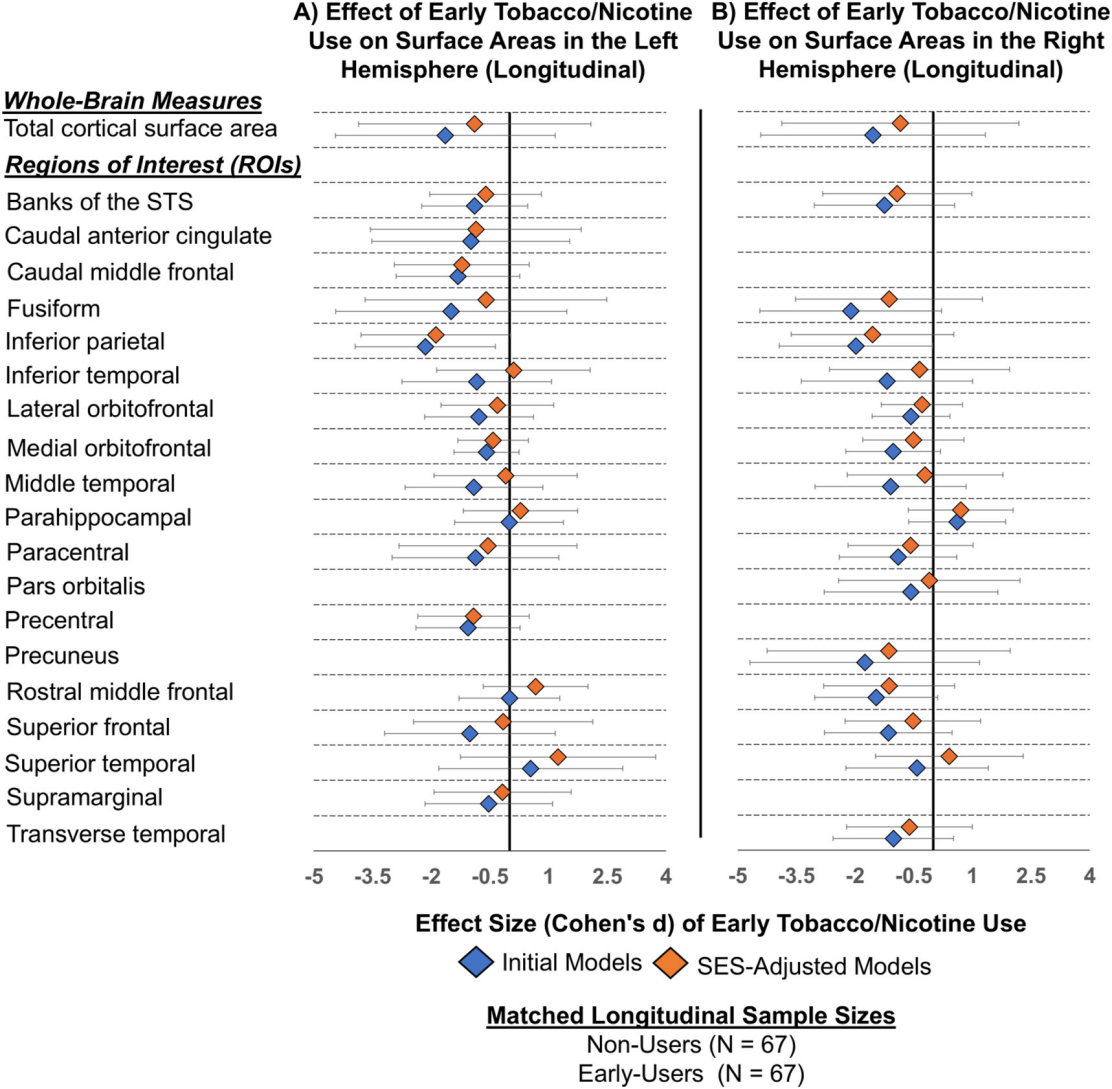
Longitudinal association between early tobacco or nicotine use and cortical surface area post propensity score matching, before and after adjustment for socioeconomic status (SES) variables. (A) Longitudinal effect sizes (Cohen’s *d*) and 95 % confidence intervals for the association between early tobacco/nicotine use and cortical surface area across regions of interest (ROIs) in the left hemisphere, before adjustment (initial models) and after adjustment for SES variables (SES-adjusted models). (B) Results for ROIs in the right hemisphere. Propensity score matching balanced early-users and non-users by age, sex, and race/ethnicity across two time points, yielding a matched sample of N=67 for longitudinal analyses. The whole-brain and ROI-based surface areas shown in the plot include only regions that were significantly associated with early tobacco/nicotine use longitudinally before propensity score matching (shown in Figure 5 and Table S6). Effect sizes were derived using linear mixed models (LMEs) and estimated marginal means. The initial model included fixed effects for early tobacco/nicotine use, ever use of other substances, pubertal development, parental monitoring, school risk/environment, and manufacturer. The child’s age, sex and race/ethnicity were not included as they were covariates in the matching process. The SES-adjusted model included all variables from the initial model, plus socioeconomic status (SES) variables: average annual family income, parental education, and economic hardships. Participant IDs were included as random effects in both models (i.e., initial and SES-adjusted). Negative effect sizes indicate smaller cortical surface areas for early-users compared to non-users. Empty spaces indicate ROIs that were not significantly associated with early tobacco/nicotine use before the propensity score matching process. Banks of the STS, banks of the superior temporal sulcus.

No significant interactions were found between early use and visit year in either the cross-sectional or longitudinal analyses (results not shown). Additionally, all effect sizes were attenuated post-SES adjustments in both the cross-sectional and longitudinal analyses. Lastly, propensity score matching revealed no significant group differences in cortical surface areas between early-users and non-users either cross-sectionally at baseline or over the two-year period (Figures 6 and 7; Table S7 and S8).

### Cortical volume and tobacco/nicotine use in relation to SES factors

Early users had smaller cortical volumes than non-users over three years, including both baseline and the 2-year follow-up, in the left Banks of the Superior Temporal Sulcus (Cohen’s *d*: −1.12, 95 % CI [−1.82 to −0.42]; p=0.047), as well as in the left (Cohen’s *d*: −1.24, 95 % CI [−1.87 to −0.61]; p<0.001) and right Middle Temporal Gyri (Cohen’s *d*: −1.06, 95 % CI [−1.71 to −0.41]; p=0.035) (Table S10). However, these longitudinal group differences were no longer significant after adjusting for SES factors, and cross-sectional effects at baseline were also not significant, regardless of SES adjustments (Tables S9–10). Additionally, there were no statistically significant interactions between early tobacco/nicotine use and visit when evaluating cortical volume (data not shown). Regardless of SES adjustments, post-hoc analyses using propensity score matching found no statistically significant group effects on cortical volume, either cross-sectionally at baseline or over the three years (baseline and 2-year follow-up) (Tables S9–S12).

### Cortical thickness and tobacco/nicotine use in relation to SES factors

Regardless of SES adjustments, no significant group differences were observed between early tobacco/nicotine users and non-users on cortical thickness, neither cross-sectionally nor longitudinally. Similarly, no effects on cortical thickness were found in our post hoc analysis using propensity score-matching (Tables S13–S16).

## Discussion

This study analyzed the impact of early tobacco/nicotine use on cognitive and brain morphometric outcomes in 9–10-year-olds, adjusting for a comprehensive set of socioeconomic status (SES) factors, including parental education, family income, and economic hardships. Without these SES-adjustments, early tobacco/nicotine use was associated with poorer cognitive scores, smaller cortical surface areas both cross-sectionally and longitudinally, and smaller cortical volumes longitudinally. These findings are consistent with prior reports [1], 33], 34], including a prior ABCD study [2]. However, the comprehensive inclusion of SES factors in our models eliminated significant negative effects of early tobacco/nicotine use on four cognitive scores and four cortical volumes that were reported in the prior ABCD study [2], which suggests an overstated negative impact of early tobacco/nicotine use on cognitive and brain measures when SES is not fully addressed.

Moreover, in contrast with findings from the earlier ABCD study [2], our longitudinal models, which included more granular SES adjustments, significantly reduced the impact of early tobacco/nicotine use on cortical surface areas in 20 brain regions. This again indicates a more substantial influence of SES on brain development than previously acknowledged. By incorporating measures of SES and economic hardship, our study suggests that SES factors may moderate or mask effects traditionally attributed to early substance use. Hence, prior reports of negative impacts of substance use on the brain might largely reflect underlying socioeconomic disadvantages.

Regardless of early tobacco/nicotine use, our results align with studies showing positive correlations between SES and brain structure, particularly surface areas, in the ABCD cohort [6], 7]. Collectively, these findings suggest that higher SES may promote more extensive gyrification, which begins in mid-gestation and continues through early postnatal brain development [35]. While gyrification starts prenatally, brain development continues well into childhood and adolescence, with SES likely exerting cumulative effects over time through multiple pathways. For example, although we focused on parental education, family income, and economic hardship, the influence of SES on cognition and brain morphometry likely extends to other factors such as nutrition, stress, and toxin exposure (e.g., lead), all crucial for brain development [36], [37], [38], [39]. These factors may shape the relationship between early tobacco/nicotine use and brain measures, emphasizing the importance of considering SES in developmental outcomes.

Preclinical studies suggest that enriched environments, analogous to higher SES, modulate the brain’s response to nicotine by influencing various neurobiological mechanisms. For example, environmental enrichment increases sensitivity to nicotine-induced locomotor activity and enhances phosphorylation of DARPP-32 and CREB in the prefrontal cortex [40]. Additionally, upregulation of microRNA-221 in the prefrontal cortex of enriched rats suppresses nicotine-induced activation of ERK and CREB signaling pathways, contributing to increased behavioral sensitivity [41]. Furthermore, enrichment enhances dopamine clearance in the medial prefrontal cortex, mitigating dysregulation of dopamine signaling associated with nicotine exposure [42]. These findings suggest that higher SES, like enriched environments, may confer neurobiological resilience, attenuating the harmful effects of early tobacco/nicotine use on brain and cognitive development. Future ABCD follow-up visits, with more detailed tobacco/nicotine usage data, could further explore whether higher SES reduces usage despite early initiation.

Our study also found that Black and Hispanic preadolescents were less likely to initiate tobacco/nicotine use than their White counterparts, despite predominantly coming from lower-income families. This aligns with previous findings of delayed initiation among Black children [43]. However, the same study also identified a crossover trend in adolescence, with tobacco use increasing among Black adolescents and decreasing among Whites [43]. Future longitudinal studies should investigate how these shifting patterns in tobacco/nicotine use, along with SES, impact brain development and health behaviors in the ABCD cohort.

### Strengths and limitations

This study utilized the robust and comprehensive longitudinal ABCD dataset, providing significant statistical power and enabling the exploration of changes over time. The diversity of the study population enhances generalizability, offering insight into how early tobacco/nicotine use impacts cognition and brain structure across different demographic and socioeconomic groups. Comprehensive assessments across multiple time points also provide a nuanced understanding of the relationship between tobacco/nicotine use, cognition, and brain morphometry.

Several limitations must be noted. First, this study focused on preadolescent children with early tobacco/nicotine use and included only one follow-up at year 3, which limited our ability to assess whether longer or persistent use might lead to cognitive decline, even after adjusting for SES [44]. Second, the reliance on self-reported data may have led to low frequencies of early use at baseline, potentially confounded or underestimated its impact on cognition and brain structure. Third, we did not account for the tobacco use severity or duration; however, we did include ‘other substances used’ as a single co-variate for all the group analyses. Lastly, while propensity score matching resulted in smaller sample sizes for both groups, potentially limiting our ability to detect more subtle associations, this approach enhanced the robustness of our findings by reducing confounds and balanced the group size.

## Conclusions

Our findings highlight the importance of incorporating granular SES adjustments in research using the ABCD and similar datasets to assess brain and cognitive outcomes. These adjustments revealed how socioeconomic conditions shape the effects of early tobacco/nicotine use. Future research should extend this work over longer follow-up periods to evaluate how SES factors influence adolescent development and explore the role of tobacco/nicotine use severity and duration in long-term outcomes. Targeted interventions addressing SES disparities could mitigate the negative effects of tobacco/nicotine use and reduce socioeconomic inequalities in public health strategies.

## Supplementary Material

Supplementary Material Details
